# Long-term results of chemoradiotherapy for stage II-III thoracic esophageal cancer in a single institution after 2000 -with a focus on comparison of three protocols-

**DOI:** 10.1186/s12885-015-1836-2

**Published:** 2015-10-27

**Authors:** Rei Umezawa, Keiichi Jingu, Haruo Matsushita, Toshiyuki Sugawara, Masaki Kubozono, Takaya Yamamoto, Yojiro Ishikawa, Maiko Kozumi, Noriyoshi Takahashi, Yu Katagiri, Noriyuki Kadoya, Ken Takeda, Hisanori Ariga, Kenji Nemoto, Shogo Yamada

**Affiliations:** 1 Department of Radiation Oncology, Tohoku University Graduate School of Medicine, 1-1, Seiryou-machi, Aobaku, Sendai, 980-8574 Japan; 2 Department of Radiological Technology, School of Health Sciences, Faculty of Medicine, Tohoku University, Sendai, Japan; 3 Department of Radiology, Iwate Medical University School of Medicine, Morioka, Japan; 4 Department of Radiation Oncology, Yamagata University School of Medicine, Yamagata, Japan

**Keywords:** Esophageal cancer, Stage II-III, Squamous cell carcinoma, Chemoradiotherapy

## Abstract

**Background:**

To evaluate the long-term results of chemoradiotherapy (CRT) for stage II-III thoracic esophageal cancer mainly by comparing results of three protocols retrospectively.

**Methods:**

Between 2000 and 2012, 298 patients with stage II-III thoracic esophageal cancer underwent CRT. Patients in Group A received two cycles of cisplatin (CDDP) at 70 mg/m^2^ (day 1 and 29) and 5-fluorouracil (5-FU) at 700 mg/m^2^/24 h (day 1–4 and 29–32) with radiotherapy (RT) of 60 Gy without a break. Patients in Group B received two cycles of CDDP at 40 mg/m^2^ (day 1, 8, 36 and 43) and 5-FU at 400 mg/m^2^/24 h (day 1–5, 8–12, 36–40 and 43–47) with RT of 60 Gy with a 2-week break. Patients in Group C received two cycles of nedaplatin at 70 mg/m^2^ (day 1 and 29) and 5-FU at 500 mg/m^2^/24 h (day 1–4 and 29–32) with RT of 60–70 Gy without a break. Differences in prognostic factors between the groups were analyzed by univariate and multivariate analyses.

**Results:**

The 5-year overall survival rates for patients in Group A, Group B and Group C were 52.4, 45.2 and 37.2 %, respectively. The 5-year overall survival rates for patients in Stage II, Stage III (non-T4) and Stage III (T4) were 64.0, 40.1 and 22.5 %, respectively. The 5-year overall survival rates for patients who received 1 cycle and 2 cycles of concomitant chemotherapy were 27.9 and 46.0 %, respectively. In univariate analysis, stage, performance status and number of concomitant chemotherapy cycles were significant prognostic factors (*p* < 0.001, *p* = 0.008 and *p* < 0.001, respectively). In multivariate analysis, stage, protocol and number of concomitant chemotherapy cycles were significant factors (*p* < 0.001, *p* = 0.043 and *p* < 0.001, respectively).

**Conclusions:**

The protocol used in Group A may be an effective protocol of CRT for esophageal cancer. It may be important to complete the scheduled concomitant chemotherapy with the appropriate intensity of CRT.

## Background

Chemoradiotherapy (CRT) for thoracic esophageal cancer has better local control and overall survival than does radiotherapy (RT) alone and is one of the curative treatments for thoracic esophageal cancer [1]. Some studies have shown that CRT for stage I esophageal cancer had a favorable treatment outcome [2, 3]. Although esophagectomy with neoadjuvant therapy has been the first choice of treatment for stage II-III, Ariga et al. and Hironaka et al. reported that treatment outcomes after CRT among patients with resectable thoracic esophageal squamous cell carcinoma were comparable to outcomes after surgery [4, 5]. A cisplatin (CDDP)-based combination as a regimen of CRT for thoracic esophageal cancer has become the standard and was used in some clinical trials [6–9]. However, the optimal schedule and dose of chemotherapy have not been established. Moreover, because techniques for radiotherapy such as intensity modulated radiation therapy have been improved, the current outcome of CRT for thoracic esophageal cancer is expected to be better than that in past trials.

We evaluated the long-term results of CRT for stage II-III thoracic esophageal cancer after 2000 mainly by comparing results of three protocols retrospectively. We also evaluated other prognostic factors that influence the results of CRT.

## Methods

### Patients

Between 2000 and 2012, 298 patients with stage II-III (T1-4 N0-1 M0: Union for International Cancer Control 2002) thoracic esophageal cancer underwent definitive CRT. This study was performed according to the principles of the Declaration of Helsinki (2013). At the time the patients gave their consent for CRT, we did not obtain comprehensive consent including future research study. Because of retrospective study, it is difficult to reacquire agreement from the patients or their family. Therefore, information disclosure is being done to give a chance of participation refusal on home page after Tohoku University School of Medicine Institutional Review Board approved this retrospective study (2014-1-543).

### Radiotherapy

Gross tumor volume was defined as the primary tumor and nodal metastasis based on upper gastrointestinal endoscopy, barium swallow, computed tomography (CT) scan and positron emission tomography (PET). If it was difficult to discriminate the primary tumor on RT planning, the clips were placed on the proximal and distal sides of the primary tumor. Initial clinical target volume (CTV) was defined as the region from the supraclavicular to celiac lymph nodes. Initial CTV was made small in consideration of the patient’s general condition. Boost CTV was defined as the primary tumor with a 20–30 mm craniocaudal margin and an approximately 5 mm radial margin and nodal metastasis. Planning target volume was defined as CTV plus a 5–15 mm margin. Basically, the initial CTV received 40 Gy at 2 Gy per day using parallel-opposed anterior-posterior fields. The boost CTV received 20–30 Gy at 2 Gy per day using parallel-oblique fields to avoid the spinal cord. In some cases, dose per fraction was set to 1.8 Gy in consideration of the patient’s general condition and the size of RT fields.

### Protocols

**Fig. 1:**
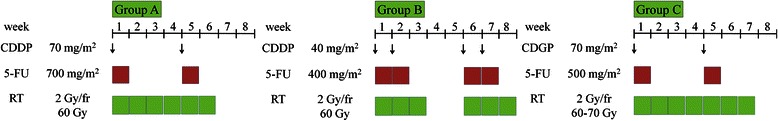
Three protocols of chemoradiotherapy for thoracic esophageal cancer in the present study. Abbreviations: CDDP, cisplatin; 5-FU, 5-fluorouracil; RT, radiotherapy; CDGP, nedaplatin

All patients underwent one of the following three protocols of CRT (Fig. 1). Adjuvant chemotherapy after CRT was performed in some patients. Patients in Group A received two cycles of chemotherapy (2-h infusion of CDDP at 70 mg/m^2^ on day 1 and continuous infusion of 5-fluorouracil [5-FU] at 700 mg/m^2^ over a 24-h period on day 1–4) with a 4-week intervals and RT dose of 60 Gy. This protocol has been performed since 2009. Patients in Group B received two cycles of chemotherapy (2-h infusion of CDDP at 40 mg/m^2^ on day 1 and 8 and continuous infusion of 5-FU at 400 mg/m^2^ over a 24-h period on day 1–5 and 8–12) with a 4-week intervals and RT dose of 60 Gy with a 2-week break after 30 Gy. This protocol has been performed mainly since 2000. Patients in Group C received two cycles of chemotherapy (2-h infusion of nedaplatin [CDGP] at 70 mg/m^2^ on day 1 and continuous infusion of 5-FU 500 mg/m^2^ over a 24-h period on day 1–5) with a 4-week interval and RT dose of 60–70 Gy. This protocol has been performed mainly since 2000. The decisions to assign patients to the three protocols was made by experienced clinicians.

### Endpoints and follow-up

The primary endpoint of the present study was the 5-year overall survival rate. The secondary endpoints were progression-free survival rate, completion rate of the protocol, pattern of the first relapse and late toxicity.

Upper gastrointestinal endoscopy, CT and PET were performed for evaluation of locoregional relapse and distant metastasis every 3–6 months. We described the first treatment at the time of the first relapse.

Late toxicities were graded according to the Common Terminology Criteria for Adverse Events version 4.0. An adverse effect more than 90 days after CRT was defined as a late toxicity.

### Statistical analysis

The characteristics of patients in Group A, Group B and Group C were compared by the 2 × 2 chi-square test for dichotomous variables or the Mann–Whitney test for continuous variables. Overall survival rate and progression-free survival rate were estimated using the Kaplan-Meier method. Differences between patient subgroups for prognostic factors were analyzed using the log-rank test as univariate analysis. Overall survival was measured from the start of RT to the date of death or last follow-up. Progression-free survival was measured from the start of RT to the date of first relapse or death due to any cause. If salvage esophagectomy was performed due to a residual lesion after CRT, we made the date of salvage esophagectomy the date of relapse. Age (66 years or less vs more than 66 years), gender (male vs female), performance status (PS) (0 vs 1 vs 2), primary site (Upper thoracic esophagus vs Middle thoracic esophagus vs Lower thoracic esophagus), stage (II vs III (non-T4) vs III (T4)), protocol (Group A vs Group B vs Group C), RT dose (60 Gy or less vs more than 60 Gy), number of concomitant chemotherapy cycles (1 cycle vs 2 cycles), and adjuvant chemotherapy (with vs without) were included in the log-rank test. Multivariate analysis was performed using the Cox proportional hazards regression model. All tests were two-sided, and statistical significance was set at the level of *p* < 0.05. Statistical analysis was performed using JMP® 10 (SAS Institute Inc., Cary, NC, USA).

## Results

The patients’ characteristics are shown in Table 1. All patients had histologically proven squamous cell carcinoma. The numbers of patients in Group A, Group B and Group C were 48, 159 and 91, respectively. There were significant differences in age, PS, stage, RT dose, number of concomitant chemotherapy cycles and adjuvant chemotherapy between the three groups (*p* < 0.001, *p* < 0.001, *p* = 0.015, *p* < 0.001, *p* = 0.019 and *p* < 0.001, respectively). The median ages of the patients in Group A, Group B and Group C were 67, 66 and 70 years, respectively. The number of patients with PS0/ PS1/ PS2 were 22/24/2, 17/120/10 and 15/57/15, respectively. The numbers of patient with stage II/ stage III (non-T4)/ stage III (T4) in Group A, Group B and Group C were 17/13/18, 47/83/29 and 30/37/24, respectively.

**Table 1:** Patients’ characteristics

Characteristic	Number of patients
Age at radiotherapy	
66 years or less	140
More than 66 years	158
Gender	
Male	255
Female	43
Performance status	
0	54
1	201
2	27
Unknown	16
Primary site	
Upper thoracic esophagus	91
Middle thoracic esophagus	160
Lower thoracic esophagus	47
Stage	
II	93
III (non-T4)	134
III (T4)	71
Protocol	
Group A	48
Group B	159
Group C	91
Radiotherapy dose	
60 Gy or less	221
More than 60 Gy	77
Concomitant chemotherapy	
1 cycle	42
2 cycles	256
Adjuvant chemotherapy	
With	67
Without	231

The completion rates of RT in Group A, Group B and Group C were 100 % (48/48), 95.0 % (151/159) and 97.5 % (89/91), respectively. Total dose at the cessation of RT was 20–64 Gy (median, 40 Gy), and a total dose of 70 Gy was planned in the prescription for 2 patients. The reasons for cessation of RT were brain infarct in 1 patient, myelosuppression in 2 patients, severe radiation pneumonia (Grade 5) in 2 patients, severe esophageal stenosis in 1 patient, esophagobronchial fistula in 1 patient, infective thrombus in 1 patient, poor general condition in 1 patient, and refusal of RT in 1 patient. The completion rates of 2 cycles of chemotherapy in Group A, Group B and Group C were 79.1 % (38/48), 91.1 % (145/159) and 80.2 % (73/91), respectively. In 19 patients, the dose intensity of chemotherapy in the second cycle was reduced due to myelosuppression and renal dysfunction. Adjuvant chemotherapy after CRT was performed in 67 patients. The number of cycles of adjuvant chemotherapy was 1–8 (median, 2). The number of patients who received adjuvant chemotherapy in Group A, Group B and Group C who received adjuvant chemotherapy were 14 (29.1 %), 45 (28.3 %) and 8 (8.8 %), respectively.

**Fig. 2:**
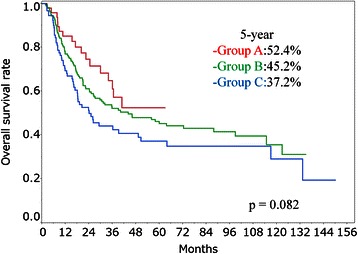
Overall survival rates for Group A, Group B and Group C

**Fig. 3:**
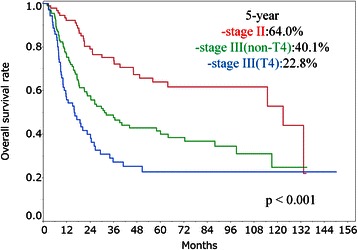
Overall survival rates for patients in stage II, stage III (non-T4) and stage III (T4)

**Fig. 4:**
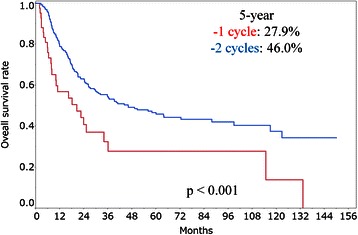
Overall survival rates for patients who received 1 cycle and 2 cycles of concomitant chemotherapy

The median follow-up period was 23.4 months (range, 1.8–150.2 months). A total of 155 patients died during the follow-up period. The 3- and 5-year survival rates in all patients were 51.5 % (95 % confidence interval [CI], 45.5–57.6) and 43.5 % (95 % CI, 37.4–50.0), respectively. Five patients died of second malignancy at 8.5–87.8 months after CRT, and 5 patients died of esophageal hemorrhage at 2.5–11.8 months after CRT. Results of the log-rank tests presented in Table 2 show the 5-year overall survival rate for each prognostic factor. The 2-year overall survival rates for patients in Group A, Group B and Group C were 74.5 % (95 % CI, 59.4–85.5), 61.1 % (95 % CI, 53.1–68.6) and 51.1 % (95 % CI, 40.4–61.8), respectively. The 5-year overall survival rates for patients in Group A, Group B and Group C were 52.4 % (95 % CI, 35.0–69.3), 45.2 % (95 % CI, 37.0–53.6) and 37.2 % (95 % CI, 26.8–48.8), respectively (Fig. 2). However, there were no significant differences between the three groups (*p* = 0.082). In univariate analysis, stage, PS and number of concomitant chemotherapy cycles were significant prognostic factors (*p* < 0.001, *p* = 0.008 and *p* < 0.001, respectively). The 5-year overall survival rates for patients in stage II, stage III (non-T4) and stage III (T4) were 64.0 % (95 % CI, 52.5–74.2), 40.1 % (95 % CI, 31.0–49.9) and 22.5 % (95 % CI, 13.7–35.5), respectively (Fig. 3). The 5-year overall survival rates for patients who received 1 cycle and patients who received 2 cycles of concomitant chemotherapy were 27.9 % (95 % CI, 14.5–46.9) and 46.0 % (95 % CI, 39.3–52.8), respectively (Fig. 4). The 5-year overall survival rates for patients with PS0, PS1 and PS2 were 48.7 % (95 % CI, 33.1–64.6), 44.3 % (95 % CI, 36.7–52.1) and 22.3 % (95 % CI, 9.5–44.1), respectively. There were no significant differences for total dose (*p* = 0.09) and adjuvant chemotherapy (*p* = 0.885). The results of multivariate analysis are shown in Table 2. Stage, protocols and number of concomitant chemotherapy cycles were significant factors (*p* < 0.001, *p* = 0.043 and *p* < 0.001, respectively). The hazard ratios (HRs) for patients in stage III (non-T4) and stage III (T4) were 2.60 (95 % CI, 1.68–4.11) and 4.17 (95 % CI, 2.47–7.12), respectively. The HRs for patients in Group B and Group C were 1.99 (95 % CI, 1.11–3.78) and 2.14 (95 % CI, 1.09–4.35), respectively. The HR with 1 cycle of concomitant chemotherapy was 3.17 (95 % CI, 1.96–5.02). We investigated overall survival rates for protocols in each stage just for reference. The 5-year overall survival rates for patients in Stage II in Group A, Group B and Group C were 77.9 % (95 % CI, 41.3–94.6), 68.3 % (95 % CI, 53.2–80.3) and 48.4 % (95 % CI, 28.7–68.7), respectively. The 5-year overall survival rates for patients in Stage III (non-T4) in Group A, Group B and Group C were 53.9 % (95 % CI, 25.5–80.0), 42.0 % (95 % CI, 30.8–54.1) and 39.2 % (95 % CI, 23.6–57.4), respectively. The 5-year overall survival rates for patients in Stage III (T4) in Group A, Group B and Group C were 25.3 % (95 % CI, 7.5–58.6), 19.1 % (95 % CI, 7.9–39.6) and 24.3 % (95 % CI, 11.0–45.4), respectively.

**Table 2:** Results of univariate and multivariate analyses

Factor	5-year OS rate (%) (95 % CI)	UA (*p* value)	MA (*p* value)
Age at radiotherapy		0.393	0.162
66 years or less	47.2 (38.7–55.9)		
More than 66 years	39.4 (30.5–49.1)		
Gender		0.100	0.215
Male	41.2 (34.6–48.1)		
Female	58.1 (40.8–73.7)		
Performance status		0.008	0.655
0	48.7 (33.1–64.6)		
1	44.3 (36.7–52.1)		
2	22.3 (9.5–44.1)		
Primary site		0.714	0.810
Upper thoracic esophagus	38.9 (24.4–55.7)		
Middle thoracic esophagus	42.9 (34.4–51.9)		
Lower thoracic esophagus	46.6 (35.7–57.8)		
Stage		<0.001	<0.001
II	64.0 (52.5–74.2)		
III (non-T4)	40.1 (31.0–49.9)		
III (T4)	22.5 (13.7–35.5)		
Protocol		0.082	0.043
Group A	52.4 (35.0–69.3)		
Group B	45.2 (37.0–53.6)		
Group C	37.2 (26.8–48.8)		
Radiotherapy dose		0.090	0.973
60 Gy or less	46.0 (28.1–51.2)		
More than 60 Gy	39.1 (38.7–53.5)		
Concomitant chemotherapy		<0.001	<0.001
1 cycle	27.9 (14.5–46.9)		
2 cycles	46.0 (39.3–52.8)		
Adjuvant chemotherapy		0.885	0.306
With	45.1 (38.1–52.5)		
Without	38.8 (26.8–52.3)		

*OS* overall survival, *CI* confidence interval, *UA* univariate analysis, *MA* multivariate analysis

The 3-year and 5-year progression-free survival rates in all patients were 35.6 % (95%CI, 30.3–41.4) and 31.2 % (95%CI, 19.6–39.9), respectively. The 5-year progression-free survival rates for patients in Group A, Group B and Group C were 46.6 % (95 % CI, 32.3–61.6), 29.0 % (95 % CI, 22.3–36.7) and 28.7 % (95 % CI, 19.6–39.9) (*p* = 0.130), respectively. The 5-year progression-free survival rates for patients in stage II, stage III (non-T4) and stage III (T4) were 47.3 % (95 % CI, 36.7–58.1), 29.5 % (95 % CI, 22.0–38.3) and 12.8 % (95 % CI, 6.2–24.7) (*p* < 0.001), respectively. The 5-year progression-free survival rates in patients with 1 cycle and 2 cycles of concomitant chemotherapy were 23.3 % (95 % CI, 12.6–39.1) and 32.7 % (95 % CI, 26.9–39.2) (*p* = 0.003), respectively. The 5-year progression-free survival rates for PS0, PS1 and PS2 were 43.1 % (95 % CI, 29.0–58.4), 29.5 % (95 % CI, 23.3–36.7) and 15.9 % (95 % CI, 5.6–37.3) (*p* = 0.032), respectively.

The patterns of the first relapse are shown in Table 3. One hundred seventy patients had relapse after CRT, and 109 patients had local relapse as the first relapse. Many of the first relapses occurred within one year after CRT. Salvage esophagectomy was performed in 31 patients who had local relapse and in 15 patients who had a residual local lesion. Salvage esophagectomy was performed in 16 patients in Stage II, 24 patients in Stage III (non-T4) and 6 patients in Stage III (T4) (Group A: 3 patients, Group B: 33 patients, Group C: 10 patients). The intervals from CRT to salvage esophagectomy were 1.5–29.2 months (median, 5.3 months). Two patients died of pneumonitis and gastric conduit necrosis after salvage esophagectomy. Salvage endoscopic mucosal resection or endoscopic submucosal dissection was performed in 12 patients who had local relapse. The intervals from CRT to salvage endoscopic therapy were 1.8–128.5 months (median, 14 months). Chemotherapy was performed in 55 patients as treatment for the first relapse after CRT and in 13 patients as treatment for relapse after salvage esophagectomy. Chemotherapy for locoregional relapse, distant metastasis and locoregional plus distant metastasis was performed in 34, 25 and 9 of the 68 patients, respectively (CDDP or CDGP + 5-FU: 29, CDGP + Taxane: 23, Taxane: 16). RT or CRT was performed in 30 patients (esophagus: 4, lymph nodes: 21, distant metastasis: 5).

**Table 3:** Pattern of first relapse

Pattern of relapse	Number of patients
Locoregional	113
(Local relapse)	(100)
Distant	41
Locoregional+distant	16
(Local relapse)	(9)

Late toxicities are shown in Table 4. Two patients died of radiation pneumonitis at 5.9 months and 10.9 months after CRT. Although those patients received steroid pulse therapy, acute exacerbation was induced after that. One patient had grade 4 radiation pneumonitis. That patient recovered after steroid pulse therapy and use of a respirator. Two patients had grade 3 radiation pneumonitis. One patient died of myocardial infarction at 114.8 months after CRT, though it was not clear whether this was caused by RT. Six patients had grade 3 cardiac disorders (heart failure: 2, acute coronary syndrome: 2, conduction disorder: 2). Grade 3 pleural effusion and pericardial effusion were detected in 2 and 4 patients, respectively. Grade 3 esophageal stenosis or fistula was detected in 5 patients. Hypothyroidsm was detected in 6 patients and they were given levothyroxine sodium hydrate.

**Table 4:** Late toxicities

Study	Grade 2	Grade 3	Grade 4	Grade 5
Radiation pneumonitis	3	2	1	2
Pleural effusion	13	2	0	0
Pericardial effusion	32	4	0	0
Heart	6	6	0	1
Skin	0	0	0	0
Esophagus	7	5	0	0
Spinal cord	0	0	0	0
Hypothyroidism	6	0	0	0

## Discussion

We discuss the results of the present study from the point of view of esophageal squamous cell carcinoma because all patients had histologically proven squamous cell carcinoma. The 3-year and 5-year survival rates after CRT for patients with stage II-III thoracic esophageal cancer including T4 in our institution were 51.5 % (95 % CI, 45.5–57.6) and 43.5 % (95 % CI, 37.4–50.0), respectively. The treatment results in the present study were better than those in previous studies, indicating that CRT for stage II-III thoracic esophageal cancer has been improved. Esophagectomy with neoadjuvant chemotherapy in Japan and esophagectomy with neoadjuvant CRT in Western countries have been the main treatments for stage II-III esophageal cancer as previously mentioned [10–12]. However, treatment results of CRT for stage II-III may be comparable to those of esophagectomy as shown in studies by Ariga et al. and Hironaka et al. [4, 5]. A meta-analysis of randomized trials in which definitive (chemo-) radiotherapy was compared with either surgery alone or surgery+/−induction treatment showed that overall survival rates after surgery and definitive CRT were similar, though there was a trend for more cancer-related deaths in the definitive CRT groups due to a higher risk of loco-regional progression [13]. Therefore, CRT is a reasonable treatment of thoracic esophageal cancer. However, the results of CRT for T4 esophageal cancer in the present study were poor, as shown in other studies [14, 15]. Those results indicate the importance of early detection of esophageal cancer.

Local relapse rates after CRT were about 30 % in some studies [4, 7, 16]. In the present study, 109 of the 298 patients had local relapse. Salvage esophagectomy has been the main curative treatment for local relapse after CRT, and salvage esophagectomy was performed in 46 patients in the present study. The large number of patients who received salvage esophagectomy may be the main reason for the better overall survival rate in the present study than the overall survival rates in previous studies. It is a fact that there were some patients with long-term survival after salvage esophageactomy. However, patients who underwent salvage esophagectomy after definitive high-dose CRT had high rates of morbidity and mortality [17]. Therefore, in the future, we may need to select patients having sensitivity to CRT for esophageal cancer more carefully.

Although local relapse has been a problem of CRT for esophageal cancer, increasing the complete response rate is an essential requirement to improve the results of CRT. Ishikura et al. reported that 3-year and 5-year overall survival rates were 63 and 51 %, respectively, for complete response patients, whereas 3-year and 5-year overall survival rates were 6 and 2 %, respectively, for non-complete response patients [18]. Therefore, it may be important to increase the treatment intensity of CRT to some extent. In the present study, the overall survival and progression-free survival rates in patients receiving two cycles of chemotherapy were better than those in patients receiving one cycle of chemotherapy between all of protocol groups, though we did not show those results. Therefore, it may be important to complete the scheduled protocol of CRT with the minimum of effort to reduce side effects. We compared the treatment results of three protocols in the present study. Since patients in Group B had a 2-week break after 30 Gy, the completion rate of CRT in Group B was the highest in the three groups. In contrast, the progression-free survival rate in Group B was lower than that in Group A. This might have been caused by protraction of RT. Protraction of RT has been shown to be detrimental in patients with head and neck cancer [19, 20]. Crehange et al. also reported that local control rate of a protocol with a 2-week break during CRT was worse than that of a protocol without a break in patients with T3N0-1 esophageal cancer [21]. Therefore, we may need to avoid unconsidered protraction of RT for esophageal cancer. CDGP used in Group C showed anti-tumor activity similar to that of CDDP and had less renal and gastrointestinal toxicity [22]. However, the treatment results in Group C were worse than those in Group A. One of those reasons might be that the general conditions of patients in Group C were poorer and they were older than those in Group A and Group B. Group A had the highest intensity of treatment in the three protocols and the protocol in Group A might be an effective CRT protocol for esophageal cancer, though the follow-up period was short and the number of patients in this group was small. Although a regimen consisting of CDDP + 5-FU and RT has been the standard for thoracic esophageal cancer, a variety of protocols of concomitant chemotherapy have been used. In the PRODIGE5/ACCORD17, definitive CRT with an FOLFOX treatment regimen (5-FU plus leucovorin and oxaliplatin) was compared with 5-FU and CDDP in patients with esophageal cancer [23]. In the Study of Chemoradiotherapy in OesoPhageal cancer with Erbitux (SCOPE) 1 trial, outcome of definitive CRT with or that without the addition of cetuximab to CDDP and 5-FU in patients with esophageal cancer were compared [24]. However, an improvement in overall survival was not achieved in either of the trials. Protocols that are superior to CDDP and 5-FU are expected to be established in the future.

Two cycles of adjuvant chemotherapy after concomitant CRT were performed in many prospective studies [4, 6–9]. Although adjuvant chemotherapy is often performed in patients with more advanced esophageal cancer, adjuvant chemotherapy had no significant benefit for overall survival rate or progression-free survival rate in the present study. Additional investigation of the effects of adjuvant chemotherapy may be necessary.

With respect to total RT dose, Intergroup (INT) 0123 carried out a clinical trial to compare standard dose RT (50.4 Gy) and high-dose RT (64.8 Gy) combined with CDDP and 5-FU [6]. They reported that there was no significant difference in median survival (13.0 vs 18.1 months) and 2-year survival (31 % vs. 40 %) between the high-dose and standard-dose groups. Therefore, a total RT dose of 50.4 Gy has often been used in CRT for esophageal cancer, though the total RT dose in the present study was 60–70 Gy. However, the treatment results may not be the same as those in previous study because RT techniques have been improving. Treatment results have in fact been different in some studies. Suh et al. reported that high-dose radiotherapy of 60 Gy or more with concurrent chemotherapy for stage II-III patients improved locoregional control and progression-free survival [25]. On the other hand, Kato et al. reported that the 1-year and 3-year overall survival rates after CRT at a dose of 50.4 Gy for stage II-III patients were 88.2 and 63.8 %, respectively, and that there were no deaths related to salvage surgery [16]. For comparison, a total dose of more than 60 Gy did not improve overall survival in the present study and might have no advantage. INT0123 also reported that 11 treatment-related deaths occurred in the high-dose group and only two deaths occurred in the standard-dose group [6]. That is one of the reasons why a total RT dose of 50.4 Gy has often been used in CRT for esophageal cancer in the U.S. The rates of late toxicities of Grade 3 or greater were 37 to 46 % in INT0123. The rate of late toxicities was 7.7 % in the present study, though those might have been underestimated because the study was a retrospective study. The rates of late toxicities in other recent studies on CRT with a total RT dose of 60 Gy were similar to that in the present study [18, 26]. Based on those results, the appropriate total RT doses for patients with esophageal cancer who will undergo salvage esophagectomy and those who will not undergo salvage esophagectomy may be 50.4 Gy and 60 Gy, respectively.

There are some limitations in the present study. First, there were significant differences in some prognostic factors between Group A, Group B and Group C. There was no defined criteria due to the retrospective analysis in the present study. If general conditions were good in less than 80 years patients without renal, cardiac and liver dysfunction, cisplatin-based regimens such as Group A and Group B tended to be performed. Therefore, selection bias may have affected outcomes of CRT in the present study. The patients in Group C were older and the general condition of patients in Group C was poorer than patients in Group A and Group B, as stated above. Second, we did not evaluate overall survival rate in view of smoking and alcohol consumption. Therefore, treatment outcomes and rate of completion of CRT might also have been affected by those factors because the patients, especially those in Group C, might have had some comorbidities caused by those factors. Third, the median follow-up period in Group A was shorter than those in Group B and Group C because the protocol for Group A has been performed since 2009. Therefore, the evaluation of 5-year overall survival rates in the three groups might be inappropriate. However, the 2-year overall survival rate in Group A was better than those in Group B and Group C, and the protocol used in Group A may therefore be an effective CRT protocol be one of for esophageal cancer.

## Conclusions

CRT for stage II-III thoracic esophageal cancer is effective, and long-term survival can be expected. However, local relapse was observed in many patients. In the future, we may need to select patients having sensitivity to CRT for esophageal cancer more carefully. The protocol used in Group A may be an effective protocol for esophageal cancer. It may be important to complete the scheduled concomitant chemotherapy with the appropriate intensity of CRT. Additional investigation is needed to improve overall survival.

## Abbreviations

CRT: Chemoradiotherapy
RT: Radiotherapy
CDDP: Cisplatin
CT: Computed tomography
PET: Positron emission tomography
CTV: Clinical target volume
5-FU: 5-fluorouracil
CDGP: Nedaplatin
PS: Performance status
HRs: Hazard ratios
SCOPE: The Study of Chemoradiotherapy in OesoPhageal cancer with Erbitux
INT: Intergroup
